# Nucleophilic Substitution at Heteroatoms—Identity Substitution Reactions at Phosphorus and Sulfur Centers: Do They Proceed in a Concerted (S_N_2) or Stepwise (A–E) Way?

**DOI:** 10.3390/molecules27030599

**Published:** 2022-01-18

**Authors:** Marian Mikołajczyk, Marek Cypryk, Bartłomiej Gostyński, Jakub Kowalczewski

**Affiliations:** 1 Department of Organic Chemistry, Centre of Molecular and Macromolecular Studies Polish Academy of Sciences, Sienkiewicza 112, 90-363 Łódź, Poland; 2 Department of Structural Chemistry, Centre of Molecular and Macromolecular Studies Polish Academy of Sciences, Sienkiewicza 112, 90-363 Łódź, Poland; bgostyns@cbmm.lodz.pl; 3 Faculty of Chemistry, Lodz University of Technology, Żeromskiego 114, 90-543 Łódź, Poland; 255338@edu.p.lodz.pl

**Keywords:** nucleophilic substitution, phosphorus, sulfur, mechanism, stereochemistry, DFT calculations

## Abstract

The mechanisms of three selected identity substitution reactions at phosphorus and sulfur occurring with stereospecific inversion have been investigated using density functional theory (DFT). The first identity reaction between methoxyl anion and methyl ethylphenylphosphinate **1** reported in 1963 has been shown to proceed in a stepwise fashion according to the addition–elimination (A–E) mechanism involving formation of a pentacoordinate phosphorus intermediate (TBI-**1**). In contrast, the results of DFT studies of the identity chloride exchange reaction in (ethoxy)ethylphosphonochloridothionate **3** in acetone solution provided evidence that it proceeds synchronously according to the classical Ingold’s S_N_2-P mechanism. DFT calculations of the methoxyl–methoxy exchange reaction at sulfur in methyl *p*-toluenesulfinate **4** catalyzed by trifluoroacetic acid in methanol revealed that it proceeds stepwise (A–E mechanism), involving the formation of the high-coordinate sulfurane intermediate. In both identity transesterification reactions, **1** and **4**, the transiently formed trigonal bipyramidal intermediates with the two methoxyl groups occupying apical positions (TBI-**1** and TBI-**4**) have higher free energy barriers for the Berry-type pseudorotation than those for direct decomposition to starting phosphinate and sulfinate ensuring stereospecific inversion of configuration at the phosphinyl and sulfinyl centers. Thus, the DFT method proved its usefulness in the distinction between both mechanisms that are often indistinguishable by kinetic measurements.

## 1. Introduction

**Scheme 1:**
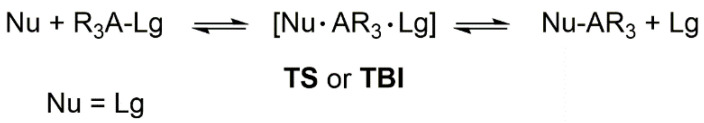
General scheme of substitution reaction.

Identity nucleophilic substitution reactions form a specific group of substitution reaction in which the nucleophilic reagent (Nu)-attacking electrophilic center (A) at carbon or heteroatoms and the leaving group (Lg) are the same (Scheme 1). The structural identity of Nu and Lg, as well as of a substrate and product, ensures the reversibility of these reactions.

The identity substitution reactions, also known as symmetric reactions, are the simplest model reactions of bimolecular nucleophilic substitutions at carbon and heteroatoms. The majority of these reactions may be carried out in aprotic solvents, and their kinetics are relatively simple because no side or catalytic reactions occur. Moreover, the relative role of bond making and breaking in the transitions state (TS) or trigonal bipyramidal intermediate (TBI) is eliminated. Therefore, for the above reasons, identity substitution reactions have become very important and attractive not only for experimental but also for theoretical chemists.

**Scheme 2:**
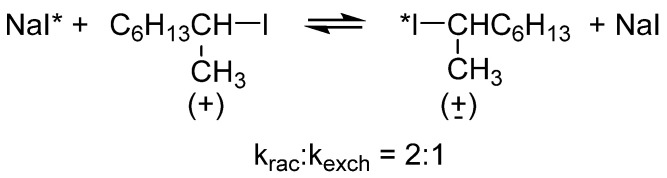
Identity iodide exchange in *d*-sec-octyl iodide.

The very early investigations on the symmetric halogen–halogen exchange reactions have contributed importantly to our present knowledge on the stereochemistry and mechanism of nucleophilic substitution reactions at saturated carbon centers. In 1935, the research group of Hughes [1] provided a direct proof of the Walden inversion by demonstration that the rate of the exchange (k_exch_) between radioactive iodide anion (*I^−^) and optically active *d*-sec-octyl iodide is equal to the rate of inversion, e.g., to half the rate of racemization (k_rac_) (Scheme 2).

**Scheme 3:**
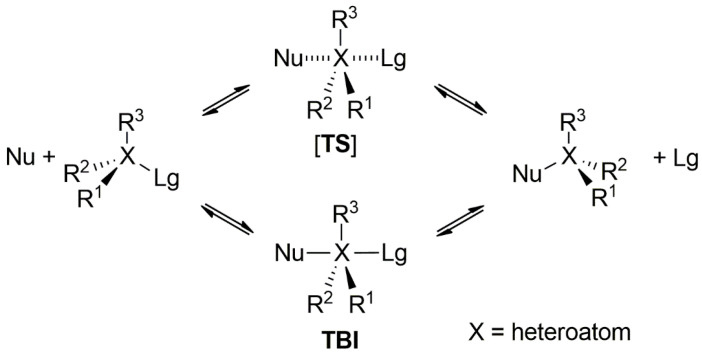
Walden inversion in substitution reactions and two possible mechanisms of substitution at heteroatoms.

In a similar way, Hughes et al. showed that stereospecific inversion occurs in the symmetric bromide exchange at carbon in optically active α-phenylethyl bromide [2] and α-bromopropionic acid [3]. The Walden inversion, observed in these halogen–halogen exchange reactions at carbon was best rationalized in terms of the classical, concerted S_N_2 mechanism with a single TS. However, it should be pointed out that the inversion of configuration observed in a substitution reaction at heteroatoms may be explained not only by the S_N_2 mechanism but also by the stepwise addition–elimination (A–E) mechanism involving the formation of a TBI and its direct decomposition to a reaction product (Scheme 3). In other words, this primary intermediate does not undergo pseudorotation—a process which changes the relative positions of substituents in trigonal bipyramid without the bond around the heteroatom breaking, and influences the stereochemical outcome of a substitution.

As these two intermediate species (TS and TBI) shown in Scheme 3 have not been experimentally detected or isolated, distinguishing between the S_N_2 and A–E mechanisms has only recently become possible. The theoretical DFT studies of nucleophilic substitution reactions for heteroatoms (P, S, Si) in simple-model symmetric and asymmetric reactions were reported at the beginning of this century by Lee and coworkers [4,5] and by the Bickelhaupt group [6,7]; they demonstrated that, for the concerted S_N_2 reactions, a double-well potential energy surface (PES) with a single TS is observed, whereas stepwise reactions (A–E) are characterized by a triple-well PES with two transition states.

**Scheme 4:**
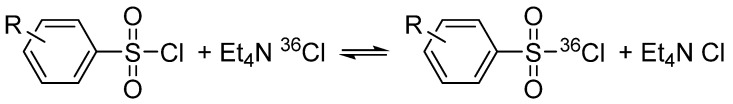
Identity chloride exchange in arenesulfonyl chlorides.

The above theoretical results agree well with our recent work [8] regarding the reactivity of arenesulfonyl chlorides and kinetics of the chloride-chloride exchange reaction at the sulfonyl center (Scheme 4). Our DFT studies showed that this reaction proceeds via a single transition state according to the S_N_2-mechanism [8]. The analogous identity fluoride exchange reaction was found to occur according to the stepwise A–E mechanism and the formation of a transient pentacoordinate sulfurane intermediate.

Being stimulated by these results, we turned our attention to the other identity substitution reactions at heteroatoms (P, S) published so far in the literature. In spite of the fact that the first identity substitution reactions at phosphorus have been reported in the last century, the fundamental question of their mechanisms has not yet been solved. Hence, the major aim of the present work was to establish by the DFT studies the relationship between the stereochemical course and the mechanism of these experimentally investigated identity reactions. Herein, we report the results of the DFT calculations on mechanisms of the three oldest identity substitution reactions occurring with inversion at the phosphinyl, thiophosphonyl and sulfinyl centers.

## 2. Results and Discussion

### 2.1. Stereochemistry and Mechanism of Identity Methoxy Exchange Reaction in Methyl Ethylphenylphosphinate ***1***

**Scheme 5:**
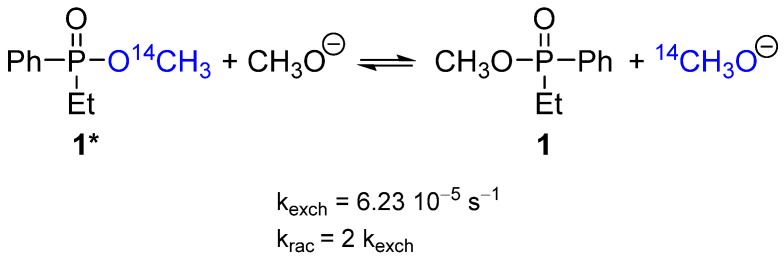
Identity methoxy exchange reaction in methyl ethylphenylphosphinate **1** (1* means **1** substituted with ^14^CH_3_).

To the best of our knowledge, the first identity substitution reaction at phosphorus proceeding with the Walden inversion was reported by Green and Hudson [9] as early as 1963. The authors investigated the symmetrical exchange of the methoxyl ion at P in methyl ethylphenylphosphinate **1**. Taking advantage of the method of Hughes [1,2,3] discussed above, they determined the rate of racemization (k_rac_) of the optically active methyl phosphinate **1** by the methoxide ion and compared this with the rate of exchange (k_exch_) of the [^14^C]methoxyl group in the radioactive ester 1* (Scheme 5).

As the pseudo-unimolecular rate of racemization of the optically active **1** has been found to be twice as large as the rate of the methoxyl exchange, the kinetic measurements provided a direct evidence that each elementary substitution step in this reaction occurs with inversion of configuration at phosphorus.

To explain the stereoinvertive course of the investigated identity reaction, Green and Hudson discussed five possible transition state (TS) structures—four having trigonal bipyramidal form and one square pyramid. According to the authors, the most probable structure is that characteristic of an S_N_2-transition state of a saturated carbon atom with the two axial bonds weaker than three basal bonds. In this place, we wish to point out that this short discussion reflected the very limited knowledge at that time on high-coordinated phosphorus compounds, the nature of bondings in P^V^-compounds as well as on the dynamic behavior of stable and transiently formed P^V^ species. At present, only two mechanistic possibilities should be considered for this identity reaction: concerted S_N_2-P and stepwise addition–elimination (A–E) mechanisms (see Scheme 3). The DFT calculations of the reaction under discussion allowed this problem to be solved definitely; they are described below.

The calculations performed to solve this question consisted of finding the appropriate stationary points (minima and transition states) of the potential energy surface (PES) for a given system, based on a relaxed scan of the reaction coordinate. In all cases, we considered the classical backside attack of the incoming nucleophile to distinguish between the plausible A–E and classical S_N_2 mechanisms.

**Figure 1:**
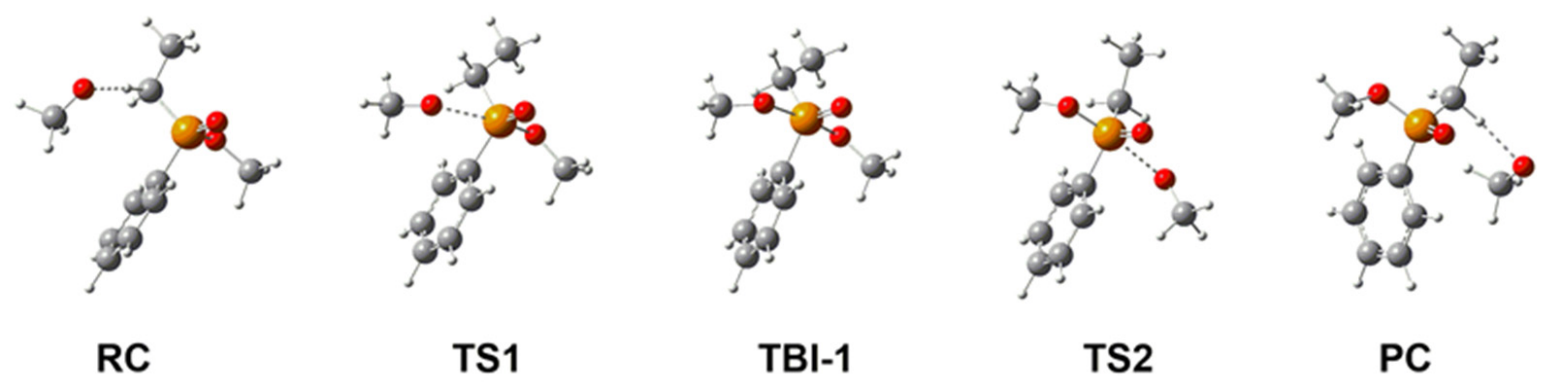
PES stationary states of identity methoxy group exchange reaction in methyl ethylphenylphosphinate **1**. Hydrogen bonds as well as forming/breaking bonds marked with dashed lines.

The outcomes of our DFT calculations carried out for the identity transesterification reaction (Scheme 5) in methanol reported by Green and Hudson [9] led us to the conclusion that it proceeds according to the A–E mechanism (Figure 1). Thus, in the first stage of the reaction, the reactants form a reactant complex (**RC**) that is transformed via a transition state (**TS1**) into the corresponding trigonal bipyramidal intermediate (**TBI-1**), with the two methoxy groups occupying apical positions. Being unstable, the latter undergoes stabilization by departure of the methoxyl ion via a twin transition state (**TS2**) to provide the product complex (**PC**). Both complexes (**RC** and **PC**), as well as transition states (**TS1** and **TS2**), are stabilized by hydrogen bonds between the methoxyl ion and the phosphinate **1**.

**Figure 2:**
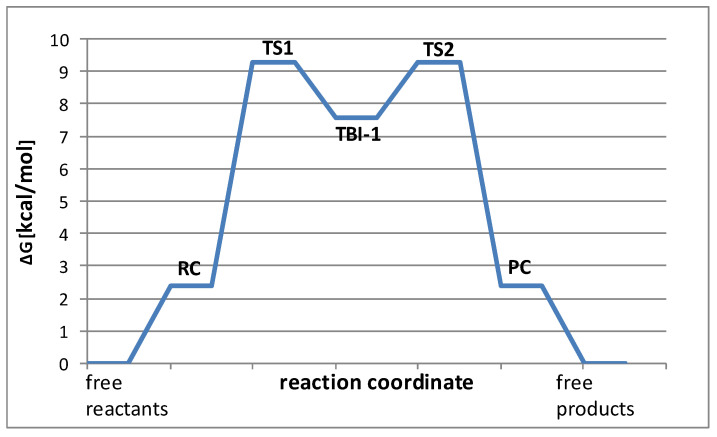
Free energy profile for the methoxy exchange reaction in methyl ethylphenylphosphinate **1** (Scheme 5).

Since the positive charge (electrophilicity) on the central phosphorus atom in the phosphinate ester **1** is the highest compared with the structurally related phosphonates and phosphates, it is reasonable to expect that the bond formation between phosphorus in the phosphinate **1** and methoxyl anion is faster than the same P-OMe bond breaking. As a consequence of this difference, the trigonal bipyramidal P^V^ intermediate (**TBI**-**1**) is transiently formed. This is in a full agreement with the fact that we were unable to find by calculation any other stable P^V^ intermediate than the one with the apical–apical MeO-P-OMe linkage. From the point of view of apicophilicity, all the five substituents at phosphorus in **TBI**-**1** are correctly placed in the trigonal bipyramidal structure. Thus, the two methoxyl groups occupy apical positions, while ethyl, phenyl and the phosphoryl oxygen are placed equatorially. For these reasons, any pseudorotation process of **TBI-1** should result in the formation of a much less stable trigonal bipyramidal P^V^ intermediate and does not occur. Therefore, instead of pseudorotation, the primary P^V^ intermediate, **TBI-1**, undergoes stabilization by departure of the methoxyl group ensuring the fully stereospecific inversion in the elementary process of transesterification. The calculated energy profile is a symmetric one as depicted in Figure 2 and overall thermochemical results are presented in Table 1.

It is worth addressing that, while being exoenergetic, the stationary points are thermodynamically unfavorable—the destabilization may result from the indispensable reorganization of the solvation shell surrounding both reactants, mainly partial desolvation of the incoming methoxide anion during the course of the reaction, which diminishes the charge delocalization and leads to increased ΔG values. The bell-curved profile is in agreement with what should be expected for a stepwise A–E mechanism [10]. Additionally, taking explicit solvent molecules into account could shift the profile towards thermodynamic stability (at least to some extent); however, such complicated and detailed calculations remain outside the scope of this general investigation.

### 2.2. Stereochemistry and Mechanism of Identity Chloride Exchange Reaction in (Ethoxy)ethylphosphonochloridothionate ***3***

**Scheme 6:**
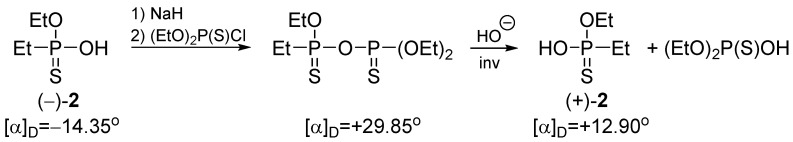
Interconversion of enantiomers of thiophosphonic acid **2**.

The stereochemistry of nucleophilic substitution reactions at phosphorus was also a main subject of our early studies. In our experimental work, the optically active (ethoxy)ethylphosphonothioic acid **2** was used as a starting material in the synthesis of a wide range of optically active phosphorus compounds. In the first place, however, the optically active thioacid **2** itself has successfully been exploited for demonstration of the Walden inversion occurring in the two-reaction sequence (Scheme 6) devised by us for the interconversion of its enantiomers [11].

**Scheme 7:**
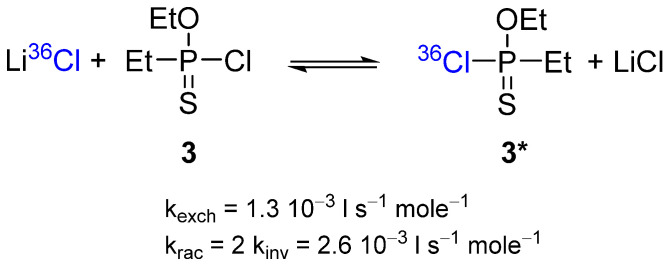
Identity chloride exchange reaction in (ethoxy)ethylphosphonochloridothionate **3** (3* means **3** substituted with ^36^Cl isotope).

Among many optically active derivatives of the thiophosphonic acid **2** prepared in our laboratory, (ethoxy)ethylphosphonochloridothionate **3** seems to be the most important. It was obtained by one of us (MM) in the reaction of the enantiomeric thioacids **2** with phosphorus pentachloride, with inversion of the configuration at the thiophosphonyl center and with almost full stereospecifity (≈98%) [12,13]. In contrast to its P(O) analogue, optically active thiophosphonyl chloride **3** was found to be optically stable and undergo slow racemization in the presence of chloride ions. These observations prompted us to investigate the chloride–chloride exchange reaction at phosphorus in **3** according to the method of Hughes to obtain another direct proof of the Walden inversion taking place in this identity reaction. Thus, the rate of racemization of the thiophosphonyl chloride (−)-**3** in the presence of lithium chloride in acetone solution was measured polarimetrically, whereas the rate of the isotopic chloride–chloride exchange was determined under the same experimental conditions (solvent, molar ratio of the reagents) for the reaction between radioactive ^36^Cl-labelled lithium chloride and racemic **3** [14] (Scheme 7).

The equality of the rate constants k_inv_ and k_exch_ proved that this reaction proceeds with the inversion of configuration at the thiophosphonyl phosphorus atom. However, the question of the mechanism of the investigated reaction remained open. As the question of the mechanism of this identity transesterification reaction could not be, and had not been, solved at that time, we decided to use the DFT calculations for assignment of the mechanism to this reaction.

**Figure 3:**
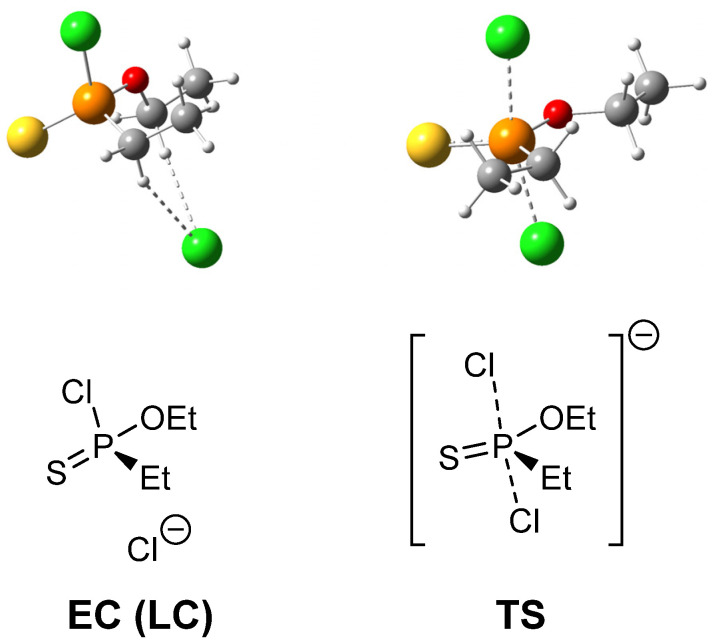
Structures of the stationary points in the chloride exchange in **3** with chloride anion.

**Figure 4:**
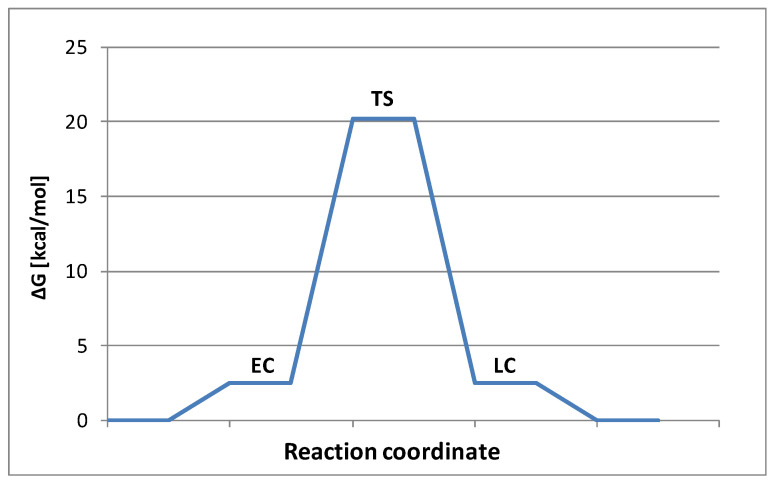
Free energy profile (kcal/mol) for the chloride exchange in **3** (Scheme 7).

Our computations confirmed that the preferred reaction route is the attack of chloride ion from the opposite side to the P-Cl bond and the ligand exchange occurs with inversion of configuration at P. The reaction path was calculated in acetone just like the original experimental measurements were made. The calculations also revealed that the reaction is a one-step process involving a single transition state (Figure 3 and Figure 4) of a geometry of distorted trigonal bipyramid, as was found previously for achiral dialkoxyphosphoryl chlorides. Relative enthalpies and free energies of the stationary points are given in Table 2. For curiosity, we have also examined the free energy profile of the side attack of chloride anion, which would result in the retention of the configuration. The free energy barrier for this route is about double that of the back attack (ΔG^‡^ = 43.4 vs. 20.2 kcal/mol for the side and back attack, respectively). Thus, our calculations confirmed that a side attack is highly unlikely.

### 2.3. Stereochemistry and Mechanism of Identity Methoxy Exchange at Sulfinyl Sulfur in Methyl p-Toluenesulfinate ***4***

**Scheme 8:**
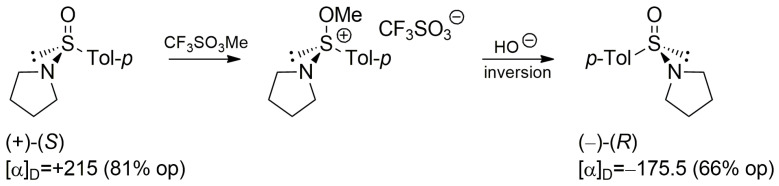
Interconversion of the enantiomers of optically active *p*-toluenosulfinylpyrrolidyne.

Our studies on the stereochemistry of organic sulfur substitution reactions at stereogenic sulfur centers started at the same time as those on chiral phosphorus compounds. At the beginning, our work was focused on the preparation and reactions of chiral sulfinyl compounds with the sulfur atom as a sole center of chirality [15]. Among the results obtained in this early stage of our investigations, two are worthy of notice because they are related to the subject of the present paper. The first concerns the interconversion of the enantiomers of optically active *p*-toluenosulfinylpyrrolidyne achieved in the two reactions sequence depicted in Scheme 8. As the first reaction (O-methylation) of (+)-(*S*)-sulfinamide does not change its configuration, the alkaline hydrolysis of the corresponding methoxy sulfonium salt formed takes place with the predominant inversion of configuration at sulfur [16].

**Scheme 9:**
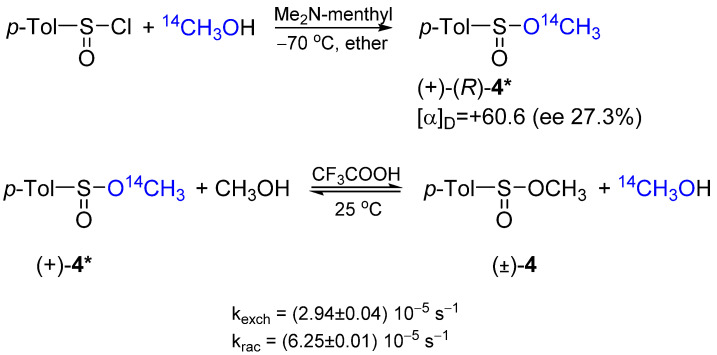
Identity methoxy exchange at sulfur in methyl *p*-toluenesulfinate **4** (4* means **4** substituted with ^14^CH_3_).

To obtain direct proof of inversion accompanying substitution at the sulfur atom, we took advantage of the classical approach used by Hughes to establish that the S_N_2-type substitution at carbon occurs with inversion. As a new and general approach to optically active aliphatic and aromatic sulfinates has been worked out in our laboratory [17], the optically active methyl *p*-toluenesulfinate 4* containing a radioactive ^14^C-methoxy group required for this study was obtained in a simple asymmetric reaction, as shown below (Scheme 9).

**Scheme 10:**
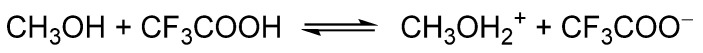
Interconversion between two equilibrium forms of trifluoroacetic acid.

**Scheme 11:**
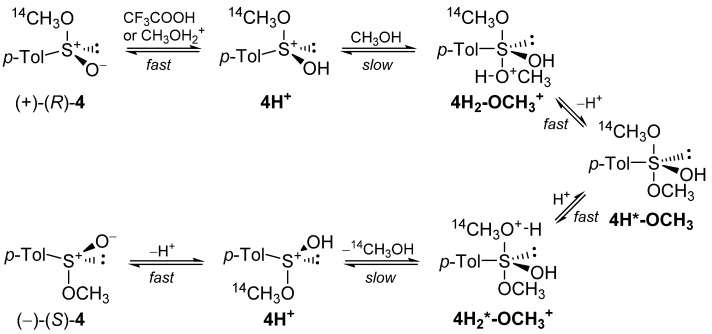
The simplified substitution route for the methanolysis of *p*-toluenesulfinate **4** leading to inversion of configuration.

Then, the rate of racemization of the methyl sulfinate (+)-(*R*)−**4** and the rate of the isotopic methoxyl–methoxy exchange in methanol in the presence of trifluoroacetic acid have been determined [18]. The results are shown in Scheme 9. The fact that the racemization is two times faster than the exchange of the radioactive methoxyl group proves unequivocally that the elementary methoxyl–methoxy substitution process occurs with net inversion of configuration at the sulfinyl center. Before presenting the results of our calculations, we first wish to propose a reasonable mechanism of the acid-catalyzed transesterification of the sulfinate **4**. It should be noted that, in a methanol solution, trifluoroacetic acid exists in a fast equilibrium, as shown below (Scheme 10):

Therefore, the free trifluoroacetic acid and protonated methanol may function as proton donors (Scheme 11).

**Figure 5:**
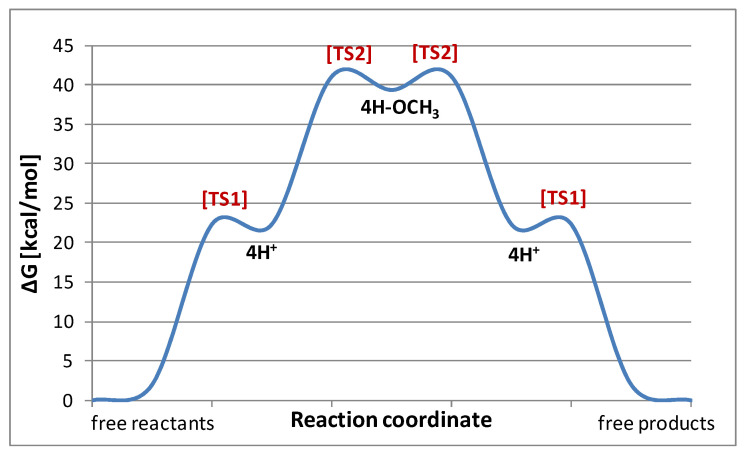
Free energy profile for reaction engaging the sulfinate ester (Scheme 11).

In the first step, the sulfinate (+)-(*R*)−**4** is protonated at the sulfinyl oxygen atom, affording the more reactive form **4H^+^** of the ester **4**. The nucleophilic attack of methanol at sulfur leads to the protonated sulfurane intermediate **4H_2_-OCH_3_^+^**, which may exist in a fast equilibrium with the dimethoxy sulfurane intermediate **4H-OCH_3_** and its two different monoprotonated forms. The diprotonated structure of both apical oxygen atoms in **4** may also be considered. However, the latter, as a much less stable compound, is not shown in Scheme 10. In the final step, the departure of radioactive methanol ^14^CH_3_OH results in the formation of the (−)-(*S*)−**4** enantiomer of the sulfinate **4**. The addition–elimination (A–E) mechanism has been taken into consideration because, in the DFT calculations, we were unable to find any transition state for this identity methoxy exchange reaction. The DFT calculations show that the **4H-OCH_3_** dimethoxy sulfurane intermediate is an energy minimum on the substitution reaction pathway. This proves that the reaction proceeds according to the addition–elimination (A–E) mechanism, despite the fact that the free energy of this intermediate is significantly higher than that of the substrates (Figure 5, Table 3). The simplified free energy profile is shown in Figure 5, where the intermediates **4H^+^** and **4H-OCH_3_** were identified. The transition states could not be localized, since they involve proton exchange with the medium which was neglected in the simplified model applied here.

## 3. Conclusions and Final Remarks

Nucleophilic substitution reactions at the stereogenic tetrahedral carbon atom proceed in a concerted way (S_N_2-C) via a transition state (TS) and are usually accompanied by Walden inversion. However, in the case of nucleophilic substitution reactions at heteroatoms (P, S, Si), which may form high-coordinate compounds, the inversion of configuration may also be observed when they proceed according to the addition–elimination (A–E) stepwise mechanism. Such a possibility was considered very early (second half of the last century) for the experimentally investigated identity substitution reactions at chiral phosphorus and sulfur, occurring stereospecifically with the Walden inversion. At the beginning of this century, theoretical chemists showed, via DFT studies, that it is possible to distinguish between the two mechanisms of simple model identity substitution reactions.

In the present work, the three selected identity substitution reactions at phosphorus and sulfur proceeding with the Walden inversion have been investigated using DFT calculations and for the first time the mechanisms of these reactions have been determined. Thus, the identity methoxyl exchange reaction in basic medium at phosphorus in methyl ethylphosphinate **1** occurs stepwise by the A–E mechanism involving transient formation of a pentacoordinate phosphorus intermediate. The second investigated reaction, the chloride–chloride exchange at phosphorus in (ethoxy)ethylthiophosphonyl chloride **3**, is a typical synchronous S_N_2-P substitution going via a TS. Finally, the acid-catalyzed methanolysis of methyl *p*-toluenosulfinate **4** (the methoxy–methoxy exchange reaction at the sulfinyl sulfur) was found to proceed stepwise (A–E mechanism) via a sulfurane intermediate. This is consistent with the common knowledge that the small methoxy anion as a good nucleophile and poor leaving group shows a tendency to form hypervalent intermediates whereas the more sterically demanding chloride anion being a poor nucleophile and good leaving group reacts preferably via a synchronous bond breaking-bond forming process.

The fact that both the identity methoxyl exchange reactions at P and S proceed stepwise by the A–E mechanism and with a full inversion of configuration was a little bit surprising, even for heteroorganic chemists, because the operation of the addition–elimination mechanism is usually connected with the pseudorotation processes of high-coordinate intermediates and racemization of optically active starting heteroatom compounds. However, it is necessary to point out that in the case of the two identity methoxyl exchange reactions investigated by us, the pentacoordinate dimethoxyphosphorane and dimethoxysulfurane intermediates have high energy barriers for pseudorotation, since the positions of all substituents at phosphorus and sulfur in trigonal bipyramidal structures are most convenient from the viewpoint of apicophilicity. Hence, they preferably undergo direct decomposition to the reaction products with stereospecific inversion. The results described here indicate that the stability of these transiently formed intermediates is one of the important factors affecting the stereochemistry–mechanism relationship of substitution reaction at heteroatoms.

## 4. Materials and Methods

### Theoretical Methods

All quantum mechanical calculations were performed using the Gaussian 16 suite of programs [19]. Geometries of the model compounds were optimized using two DFT methods: the hybrid B3LYP density functional [20] corrected for dispersion interactions using Grimme GD3 empirical term [21], with the Def2TZVP basis set [22] in methanol or acetone solution. SCRF calculations in methanol or acetone were performed using the CPCM model with UFF atomic radii, as implemented in Gaussian 16 [19]. All stationary points were identified as stable minima and transition states by frequency calculations. The vibrational analysis provided thermal enthalpy and entropy corrections at 298 K within the rigid rotor/harmonic oscillator/ideal gas approximation [19]. Thermochemical corrections were scaled by a factor of 0.99. Details are given in the Supplementary Materials.

## Figures and Tables

**Table 1:** Gibbs free energies (kcal/mol) relative to the sum of the free energies of free substrates for the reaction in Scheme 5.

	ΔH	ΔG
**RC**	−6.5	2.4
**TS1**	−2.7	9.3
**TBI-1**	−4.3	7.6
**TS2**	−2.7	9.3
**PC**	−6.5	2.4

**Table 2:** Gibbs free energies (kcal/mol) of the stationary points in chloride exchange in **3** (Scheme 7) relative to the sum of the free energies of free substrates.

	ΔH	ΔG
**EC**	−3.6	2.5
**TS**	12.5	20.2

**Table 3:** Gibbs free energies (kcal/mol) relative to the sum of the free energies of free substrates for the reaction in Scheme 11.

		ΔH	ΔG
TolS(O)OMe + CF_3_COOH + MeOH	free reactants	0.0	0.0
TolS(OH^+^)OMe + CF_3_COO^(−)^ + MeOH	**4-H^+^**	22.0	22.3
TolS(OH)(OMe)_2_ + CF_3_COOH	**4-OMe**	26.0	39.3

## Data Availability

Data is contained within the article and Supplementary Materials.

## Supplementary Materials

The following supporting information can be downloaded online. Cartesian coordinates of all calculated structures as well as their enthalpy, Gibbs free energy and entropy values and the imaginary frequencies for the transition states.

## Sample Availability

Samples of the compounds are not available from the authors.
