# Correction: Mikołajczyk et al. Nucleophilic Substitution at Heteroatoms—Identity Substitution Reactions at Phosphorus and Sulfur Centers: Do They Proceed in a Concerted (SN2) or Stepwise (A–E) Way? *Molecules* 2022, *27*, 599

**DOI:** 10.3390/molecules27123820

**Published:** 2022-06-14

**Authors:** Marian Mikołajczyk, Marek Cypryk, Bartłomiej Gostyński, Jakub Kowalczewski

**Affiliations:** 1 Department of Organic Chemistry, Centre of Molecular and Macromolecular Studies Polish Academy of Sciences, Sienkiewicza 112, 90-363 Łódź, Poland; 2 Department of Structural Chemistry, Centre of Molecular and Macromolecular Studies Polish Academy of Sciences, Sienkiewicza 112, 90-363 Łódź, Poland; bgostyns@cbmm.lodz.pl; 3 Faculty of Chemistry, Lodz University of Technology, Żeromskiego 114, 90-543 Łódź, Poland; 255338@edu.p.lodz.pl

(1) The authors would like to correct mistakes in the title paper [1]. The wrong central atom, P (phosphorus), in Scheme 8 has been corrected to S (sulfur), and the anion in the intermediate ion pair has been changed to CF_3_SO_3_^−^. The corrected Scheme 8 is presented below. 

(2) Moreover, in the line below Scheme 9, the word ‘sulfonate’ has been corrected to ‘sulfinate’. The correct sentence should be as follows:

‘Then, the rate of racemization of the methyl **sulfinate** (+)-(*R*)−**4** and the rate of the isotopic methoxyl–methoxy exchange in methanol in the presence of trifluoroacetic acid have been determined [18]’.

(3) In the References section the title of reference [17] contains the misspelled word: “Stom”. It should be: “Atom”.

The authors apologize for any inconvenience caused and state that the scientific conclusions are unaffected. The original publication has also been updated.

## Figures and Tables

**Scheme 8:**
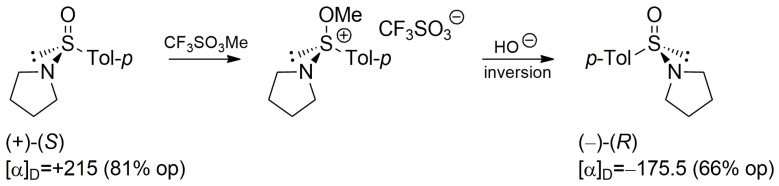
Interconversion of the enantiomers of optically active *p*-toluenosulfinylpyrrolidyne.
