# Supplementary material for: Nucleophilic Substitution at Heteroatoms—Identity Substitution Reactions at Phosphorus and Sulfur Centers: Do They Proceed in a Concerted (SN2) or Stepwise (A–E) Way?
Source: Molecules. 2022 Jan 18;27(3):599. doi: 10.3390/molecules27030599 (PMC8839028; doi:10.3390/molecules27030599)
Supplement: Supplementary file 1 [file molecules-27-00599-s001.zip › molecules-1516785-supplementary.pdf]

Nucleophilic Substitution at Heteroatoms. Identity Substitution Reactions at Phosphorus and Sulfur Centres: Do They Proceed in a Concerted (SN2) or Stepwise (A-E) Way?

Marian Mikołajczyk, Marek Cypryk, Bartłomiej Gostyński, Jakub Kowalczewski

Supplementary Information

Tables S1-S7. B3LYP-GD3/Def2TZVP geometries of species engaged in the identity methoxy exchange reaction in methyl ethylphenylphosphinate (in Cartesian coordinates).

- Table S1 – geometry of PhEtP(O)(OMe) in acetone

Element	X [Å]	Y [Å]	Z [Å]
P	1.146728	0.062031	0.495412
O	1.486493	-0.305928	1.892113
O	1.597113	1.581429	0.224989
C	1.995736	-0.933294	-0.755309
H	1.770213	-0.538519	-1.748082
H	3.061026	-0.758394	-0.582447
C	1.662884	-2.426359	-0.674981
H	2.232183	-2.978318	-1.423522
H	1.910498	-2.832180	0.306335
H	0.602990	-2.607799	-0.859476
C	1.323592	2.315498	-0.981417
H	1.706623	3.320486	-0.823211
H	1.832051	1.866741	-1.836148
H	0.250711	2.361472	-1.172726
C	-0.631478	-0.020508	0.166610
C	-1.505109	0.013308	1.257467
C	-1.156150	-0.101673	-1.127175
C	-2.879507	-0.025433	1.057701
H	-1.1620	0.060371	2.2617
C	-2.531501	-0.142921	-1.324587
H	-0.501825	-0.138616	-1.988252
C	-3.393788	-0.102782	-0.233394
H	-3.548556	-0.20	1.908253
H	-2.928562	-0.208257	-2.329314
H	-4.464583	-0.136461	-0.388919

- Table S2 – geometry of methoxyl ion(CH_3O^-) in acetone

Element	X [Å]	Y [Å]	Z [Å]
O	-0.802775	0.27	-0.20
C	0.552779	-0.87	0.06
H	1.035380	0.362832	-0.950478
H	1.035097	0.642205	0.789322
H	1.035051	-1.4733	0.161279

- Table S3 – geometry of **PC** in acetone

Element	X [Å]	Y [Å]	Z [Å]
P	-1.118595	-0.366955	0.309601
O	-1.512365	-0.350638	1.745552
O	0.126270	3.348597	0.504580
C	-1.614670	1.064833	-0.649046
H	-1.270181	0.909153	-1.674283
H	-1.039164	1.910470	-0.220457
C	-3.1270	1.344419	-0.603814
H	-3.344611	2.254134	-1.163634
H	-3.460717	1.494026	0.422171
H	-3.703378	0.530373	-1.037934
C	1.140324	3.228498	-0.398296
H	1.334145	4.146306	-1.012616
H	0.987650	2.421435	-1.160854
H	2.139559	2.984710	0.046421
C	0.668342	-0.573951	0.102952
C	1.515441	-0.385818	1.195547
C	1.214669	-0.892943	-1.143270
C	2.892344	-0.507650	1.043926
H	1.089905	-0.140425	2.159644
C	2.590129	-1.017627	-1.293659
H	0.564923	-1.049083	-1.995484
C	3.430130	-0.822896	-0.2020
H	3.544581	-0.357562	1.894908
H	3.7261	-1.266417	-2.261209
H	4.502224	-0.917773	-0.318186
C	-1.634302	-2.930343	-0.072692
H	-2.047152	-3.034569	0.931349
H	-0.585072	-3.233749	-0.070617
H	-2.191041	-3.557213	-0.765258
O	-1.769410	-1.576536	-0.538601

- Table S4 – geometry of **RC** in acetone

Element	X [Å]	Y [Å]	Z [Å]
P	1.118595	-0.366955	0.309601
O	1.512365	-0.350638	1.745552
O	-0.126270	3.348597	0.504580

C	1.614670	1.064833	-0.649046
H	1.270181	0.909153	-1.674283
H	1.039164	1.910470	-0.220457
C	3.1270	1.344419	-0.603814
H	3.344611	2.254134	-1.163634
H	3.460717	1.494026	0.422171
H	3.703378	0.530373	-1.037934
C	-1.140324	3.228498	-0.398296
H	-1.334145	4.146306	-1.012616
H	-0.987650	2.421435	-1.160854
H	-2.139559	2.984710	0.046421
C	-0.668342	-0.573951	0.102952
C	-1.515441	-0.385818	1.195547
C	-1.214669	-0.892943	-1.143270
C	-2.892344	-0.507650	1.043926
H	-1.089905	-0.140425	2.159644
C	-2.590129	-1.017627	-1.293659
H	-0.564923	-1.049083	-1.995484
C	-3.430130	-0.822896	-0.2020
H	-3.544581	-0.357562	1.894908
H	-3.7261	-1.266417	-2.261209
H	-4.502224	-0.917773	-0.318186
C	1.634302	-2.930343	-0.072692
H	2.047152	-3.034569	0.931349
H	0.585072	-3.233749	-0.070617
H	2.191041	-3.557213	-0.765258
O	1.769410	-1.576536	-0.538601

- Table S5 - geometry of **TBI-1** in acetone

Element	X [Å]	Y [Å]	Z [Å]
P	-0.961237	0.092559	-0.4567
O	-1.422655	-0.114274	-1.874181
O	-0.647801	1.891093	-0.765706
C	-2.129602	0.594854	0.899579
H	-1.511229	0.880958	1.755598
H	-2.578819	1.519324	0.537103
C	-3.230751	-0.365408	1.346510
H	-3.912599	0.149228	2.030551
H	-3.818392	-0.718599	0.497058

H	-2.8288	-1.239787	1.852038
C	-0.171945	2.785738	0.202439
H	0.244792	3.670860	-0.294919
H	-0.962041	3.138770	0.884178
H	0.627111	2.363504	0.830142
C	0.834460	-0.035417	-0.034866
C	1.786714	0.1564	-1.036638
C	1.278539	-0.338989	1.252738
C	3.146289	0.037389	-0.764125
H	1.458250	0.403231	-2.038046
C	2.638364	-0.432303	1.537932
H	0.557201	-0.515049	2.040588
C	3.578489	-0.250246	0.528021
H	3.869557	0.174756	-1.559304
H	2.963034	-0.654911	2.547502
H	4.636412	-0.333208	0.744803
C	-0.604701	-2.635929	-0.649871
H	-1.015756	-2.653076	-1.666153
H	0.490899	-2.575676	-0.737176
H	-0.845357	-3.587167	-0.162808
O	-1.143498	-1.5930	0.114829

- Table S6 - geometry of **TS1** in acetone

Element	X [Å]	Y [Å]	Z [Å]
P	-1.5703	-0.186188	-0.396693
O	-1.361622	-0.352208	-1.836097
O	-0.168144	2.126828	-1.015041
C	-2.039840	0.906248	0.630738
H	-1.426865	1.254166	1.4645
H	-2.209537	1.775082	0.0174
C	-3.354381	0.305371	1.134516
H	-3.938437	1.078592	1.639145
H	-3.958715	-0.081513	0.311617
H	-3.186940	-0.510523	1.835753
C	0.283019	3.0596	-0.065961
H	-0.516731	3.416927	0.595170
H	1.023534	2.556871	0.641580
H	0.793756	3.895283	-0.489780

C	0.768097	-0.169821	-0.031620
C	1.682488	-0.408386	-1.060794
C	1.242471	-0.036214	1.274207
C	3.041847	-0.494506	-0.792108
H	1.317962	-0.518135	-2.073568
C	2.607422	-0.1147	1.543529
H	0.550807	0.118975	2.092667
C	3.511280	-0.343481	0.513098
H	3.739360	-0.680215	-1.599986
H	2.960866	-0.0199	2.561090
H	4.571852	-0.408644	0.722026
C	-0.893624	-2.831361	-0.075389
H	-1.152660	-2.984202	-1.125740
H	0.193990	-2.886996	0.032198
H	-1.349230	-3.618378	0.525093
O	-1.397753	-1.586713	0.4844

- Table S7 –geometry of **TS2** in acetone

Element	X [Å]	Y [Å]	Z [Å]
P	1.5703	-0.186188	-0.396693
O	1.361622	-0.352208	-1.836097
O	0.168144	2.126828	-1.015041
C	2.039840	0.906248	0.630738
H	1.426865	1.254166	1.4645
H	2.209537	1.775082	0.0174
C	3.354381	0.305371	1.134516
H	3.938437	1.078592	1.639145
H	3.958715	-0.081513	0.311617
H	3.186940	-0.510523	1.835753
C	-0.283019	3.0596	-0.065961
H	0.516731	3.416927	0.595170
H	-1.023534	2.556871	0.641580
H	-0.793756	3.895283	-0.489780
C	-0.768097	-0.169821	-0.031620
C	-1.682488	-0.408386	-1.060794
C	-1.242471	-0.036214	1.274207
C	-3.041847	-0.494506	-0.792108
H	-1.317962	-0.518135	-2.073568

C	-2.607422	-0.1147	1.543529
H	-0.550807	0.118975	2.092667
C	-3.511280	-0.343481	0.513098
H	-3.739360	-0.680215	-1.599986
H	-2.960866	-0.0199	2.561090
H	-4.571852	-0.408644	0.722026
C	0.893624	-2.831361	-0.075389
H	1.152660	-2.984202	-1.125740
H	-0.193990	-2.886996	0.032198
H	1.349230	-3.618378	0.525093
O	1.397753	-1.586713	0.4844

- Table S8 – B3LYP-GD3/Def2TZVP calculated values of the thermodynamical potentials for the structures considered in tables S1-S7, with imaginary frequency values for transition states.

structure	H [hartree]	G [hartree]	S [cal/mol·K]	Im. Freq [cm^{-1}]
PhEtP(O)(OMe)	-842.697883	-842.751830	113.541	N/A
CH_3O^-	-115.221878	-115.247991	54.957	N/A
**PC**	-957.930152	-957.995985	138.557	N/A
**RC**	-957.930152	-957.995985	138.557	N/A
**TBI-1**	-957.926668	-957.987741	128.540	N/A
**TS1**	-957.924025	-957.985001	128.335	-55.55
**TS2**	-957.924025	-957.985001	128.335	-55.55

Tables S9-S11. B3LYP-GD3/Def2TZVPgeometries of species engaged in the identity chloride exchange reaction in (ethoxy)ethylphosphonochloridothionate (in Cartesian coordinates).

- Table S9 – geometry of EtP(S)(OEt)Cl in acetone

Element	X [Å]	Y [Å]	Z [Å]
P	0.511368	-0.091575	0.076126
S	1.824834	-0.048324	1.488037
Cl	0.455411	-1.895965	-0.963163
O	-0.966834	0.062853	0.639345
C	0.715010	1.098235	-1.279703
H	-0.050556	0.877470	-2.0264
H	1.682463	0.874088	-1.731260
C	-2.172257	0.088630	-0.185971
H	-2.166910	-0.786947	-0.836235
H	-2.151136	0.989562	-0.8760
C	-3.3622	0.082770	0.745156
H	-4.279079	0.1134	0.157438
H	-3.365374	-0.819803	1.356695
H	-3.341551	0.954045	1.4401
C	0.639197	2.550310	-0.802361
H	1.418979	2.766302	-0.072380
H	0.773192	3.217334	-1.654339
H	-0.326779	2.773649	-0.347756

- Table S10 – geometry of **EL/LC** in acetone

Element	X [Å]	Y [Å]	Z [Å]
P	0.937160	-0.129657	0.259255
S	1.1188	-0.080179	2.187039
Cl	-3.511514	-0.923364	0.326058
O	0.3193	1.163194	-0.436426
C	0.041960	-1.557947	-0.398874
H	-0.941744	-1.498709	0.082671
H	0.551477	-2.442985	-0.013884
C	-0.912556	1.777953	0.073020
H	-0.687015	2.214454	1.046530
H	-1.674127	1.4134	0.191081
C	-1.337362	2.822682	-0.929336

H	-2.244559	3.313823	-0.572531
H	-0.562268	3.579149	-1.059546
H	-1.550964	2.366108	-1.896561
C	-0.111977	-1.593634	-1.918055
H	0.851587	-1.674694	-2.421093
H	-0.715620	-2.458272	-2.196789
H	-0.617899	-0.699459	-2.282322
Cl	2.746839	-0.163442	-0.751243

- Table S11 – geometry of **TS** in acetone

Element	X [Å]	Y [Å]	Z [Å]
P	0.294415	0.109473	-0.211249
S	1.428837	0.498518	-1.728123
Cl	-0.538456	2.501337	0.1668
O	-1.211675	-0.283762	-0.494429
C	0.806623	0.123524	1.563606
H	0.420429	-0.796216	1.995050
H	0.318875	0.974899	2.026656
C	-2.158421	-0.576311	0.569696
H	-2.167911	0.267171	1.261830
H	-1.816071	-1.473555	1.087973
C	-3.5188	-0.782612	-0.070240
H	-4.246041	-1.012053	0.702299
H	-3.828108	0.1185	-0.596484
H	-3.479413	-1.612243	-0.777412
C	2.322767	0.206293	1.710864
H	2.714270	1.127761	1.279719
H	2.573967	0.193333	2.772923
H	2.811827	-0.639859	1.230132
Cl	0.794261	-2.402567	-0.092546

- Table S12 – B3LYP-GD3/Def2TZVP calculated values of the thermodynamical potentials for the structures considered in tables S9-S11, with imaginary frequency values for transition states.

structure	H [hartree]	G [hartree]	S [cal/mol·K]	Im. Freq [cm^{-1}]
EtP(S)(OEt)Cl	-1433.557135	-1433.607163	105.293	N/A
Cl^-	-460.398371	-460.415754	36.586	N/A

**EL/LC**	-1893.961314	-1894.019311	122.065	N/A
**TS**	-1893.935682	-1893.991097	116.629	-228.

Tables S13-S18. B3LYP-GD3/Def2TZVPgeometries of species engaged in the identity methoxy exchange at sulfinyl sulfur in methyl *p*-toluenesulfinate (in Cartesian coordinates).

- Table S13– geometry of TolS(O)(OMe) in acetone

Element	X [Å]	Y [Å]	Z [Å]
C	-0.642553	-1.067435	0.577540
C	-0.019840	0.138182	0.260943
C	-0.754187	1.214454	-0.213094
C	-2.125822	1.072805	-0.397510
C	-2.773134	-0.126943	-0.101074
C	-2.9645	-1.193170	0.390257
H	-0.065390	-1.9619	0.958909
H	-0.254592	2.147298	-0.438158
H	-2.699888	1.910857	-0.7735
H	-2.496321	-2.130907	0.630216
C	-4.255753	-0.285677	-0.299455
H	-4.465099	-1.045881	-1.0561
H	-4.740166	-0.610451	0.623936
H	-4.719194	0.6471	-0.619141
S	1.739793	0.325415	0.562322
O	2.102088	1.694223	0.139113
O	2.160739	-0.731816	-0.639690
C	3.552027	-1.122717	-0.621389
H	3.840694	-1.490617	0.365422
H	3.643975	-1.923084	-1.351294
H	4.190111	-0.286581	-0.910241

- Table S14– geometry of **4-H**$^+$ in acetone

Element	X [Å]	Y [Å]	Z [Å]
C	0.545592	-1.126171	-0.072428
C	0.041674	0.1735	-0.168331
C	0.885109	1.281991	-0.197833
C	2.251079	1.082062	-0.090815
C	2.789041	-0.203114	0.027852
C	1.916609	-1.295562	0.032415
H	-0.113643	-1.981025	-0.070433

H	0.489693	2.283691	-0.296091
H	2.909944	1.940535	-0.103899
H	2.315286	-2.298251	0.117248
C	4.269869	-0.403540	0.171111
H	4.549334	-0.381010	1.228202
H	4.581339	-1.367593	-0.230413
H	4.828470	0.385077	-0.332723
S	-1.684415	0.448217	-0.417609
O	-1.986132	1.707176	0.504260
O	-2.234267	-0.830829	0.327842
C	-3.619687	-1.237278	0.031946
H	-3.780262	-1.254683	-1.043575
H	-3.695406	-2.235044	0.449923
H	-4.303510	-0.554092	0.528203
H	-1.543127	1.669609	1.374984

- Table S15– geometry of **4-OMe** in acetone

Element	X [Å]	Y [Å]	Z [Å]
S	1.697696	0.014311	-0.283247
O	1.799556	-1.668892	-0.635088
C	1.305741	-2.689294	0.237459
H	1.314010	-3.620355	-0.329791
H	1.944255	-2.792586	1.115925
H	0.282507	-2.481623	0.561220
C	-0.093492	0.2947	-0.1037
C	-0.852256	-0.417572	-1.187054
C	-0.706875	0.422804	1.070521
C	-2.239682	-0.409741	-1.092907
H	-0.369728	-0.760256	-2.092523
C	-2.090790	0.405755	1.157033
H	-0.109695	0.759187	1.905704
C	-2.880777	-0.2627	0.077153
H	-2.829010	-0.734026	-1.941874
H	-2.567480	0.718304	2.078440
C	-4.381813	0.023458	0.174556
H	-4.842535	-0.603921	-0.588409
H	-4.720131	-0.317755	1.154156
H	-4.757319	1.041481	0.038776
O	2.289466	-0.254251	1.217378

H	2.417253	0.632405	1.598375
O	1.749892	1.804777	0.266095
C	1.366585	2.679990	-0.766615
H	0.278537	2.824373	-0.794613
H	1.843657	3.650690	-0.609732
H	1.681390	2.307725	-1.753622

- Table S16– geometry of CF_3COOH in acetone

Element	X [Å]	Y [Å]	Z [Å]
C	-0.595641	-0.1578	0.29
C	1.519763	-1.039498	0.86
O	-1.1576	-0.678181	1.086267
O	-1.1305	-0.676210	-1.087557
F	-1.187971	1.191628	0.1055
F	0.950129	0.157606	0.0135
F	1.493087	1.223469	0.64
H	2.487935	-0.943060	-0.68

- Table S17– geometry of CF_3COO^- in acetone

Element	X [Å]	Y [Å]	Z [Å]
C	-0.526120	0.013739	-0.1712
C	1.049671	0.012315	-0.4676
O	1.590771	1.128730	-0.2454
O	1.532390	-1.133273	-0.2414
F	-1.015995	-0.573899	1.120454
F	-1.029618	-0.682630	-1.051695
F	-1.079565	1.243197	-0.060173

- Table S18– geometry of MeOH in acetone

Element	X [Å]	Y [Å]	Z [Å]
C	-0.669370	0.019642	0.00
H	-1.026652	0.547242	-0.89023
H	-1.090184	-0.985392	-0.0366
H	-1.026589	0.546513	0.890653
O	0.751826	-0.122877	-0.14
H	1.145032	0.756797	0.58

- Table S19 – B3LYP-GD3/Def2TZVP calculated values of the thermodynamical potentials for the structures considered in tables S13-S18, with imaginary frequency values for transition states.

**structure**	**H [hartree]**	**G [hartree]**	**S [cal/mol·K]**	**Im. Freq [cm^{-1}]**
TolS(O)(OMe)	-859.541098	-859.594965	113.371	N/A
**4-H$^+$**	-859.939516	-859.992490	111.493	N/A
**4-OMe**	-975.226724	-975.286583	125.985	N/A
CF_3COOH	-526.998371	-527.036904	81.100	N/A
CF_3COO^-	-526.564902	-526.603773	81.810	N/A
MeOH	-115.727304	-115.754388	57.200	N/A
